# Analysis of the effect of phloroglucinol on pregnancy outcomes involving frozen embryo transfers in patients with endometriosis: A retrospective case-control study

**DOI:** 10.3389/fsurg.2022.994775

**Published:** 2023-01-06

**Authors:** Wen-Juan Pang, Xu Feng, Xiang Wang, Liang Wang, Ning-Xia Sun

**Affiliations:** Reproductive Medicine Center, Second Affiliated Hospital of Naval Medical University, Shanghai, China

**Keywords:** frozen embryo transfer, phloroglucinol, endometriosis, endometrial peristalsis wave, pregnancy outcome

## Abstract

**Objective:**

Abnormal contraction of uterus and vascular smooth muscle lead to the formation of hypoxia environment in uterus. Abnormal contraction may be the basis of dysmenorrhea, endometriosis, infertility and other diseases. Phloroglucinol is a non-atropine and non-papaverine smooth muscle spasmolytic agent, which can reduce the abnormal contraction of uterine smooth muscle. This study investigated the effect of phloroglucinol on frozen embryo transfer in patients with endometriosis.

**Methods:**

The data of patients with endometriosis who underwent a frozen embryo transfer in Shanghai Changzheng Hospital from August 2018 to August 2021, comprising a total of 453 cycles, were retrospectively analyzed. The patients for whom phloroglucinol was included over 217 cycles were administered intramuscully 40 mg phloroglucinol starting on the day of progesterone administration, then once daily up to day 7 after the embryo transfer. Those for whom phloroglucinol was not administered over 236 cycles were used as the control group. The age of 35 years was used as a boundary in this study to observe the pregnancy outcomes of patients in the two different age groups.

**Results:**

The biochemical pregnancy rate (63.13% vs. 51.27%), embryo implantation rate (44.64% vs. 33.60%), clinical pregnancy rate (59.64% vs. 48.30%), and live birth rate (52.99% vs. 36.86%) after the administration of phloroglucinol were higher than for patients in the control group, and the early abortion rate (7.75% vs. 20.18%) was also lower. The differences were statistically significant (*P* < 0.05). In particular, in the age group <35 years old, the embryo implantation rate (51.81% vs. 39.38%), clinical pregnancy rate (69.34% vs. 57.55%), and the live birth rate (63.50% vs. 44.60%) after phloroglucinol intervention rose significantly, and the abortion rate dropped (6.32% vs. 17.5%), indicating a statistically significant difference (*P* < 0.05). However, pregnancy outcomes showed no difference in the age group ≥35 years old (*P* > 0.05).

**Conclusion:**

Continuous low-dose phloroglucinol pretreatment before and after frozen embryo transfer can improve both the clinical pregnancy and live birth rates and reduce the risk of abortion in younger infertile patients with endometriosis.

## Introduction

With the accelerated pace of work and changes in living environments, the incidence of infertility is increasing annually. According to information released by the Department of Maternal and Child Health, National Health Commission, the incidence of infertility of men and women in China is 7%–10% (1). Several causes have been linked to infertility. Studies have shown that 25%–50% of infertile women suffer from endometriosis, 30%–50% of whom also suffer from infertility (2). Moreover, the implantation and pregnancy rates of *in vitro* fertilization (IVF) in endometriosis-related infertile patients are lower than those in tubal factor infertility patients (3). This may be related to the damage of endometrial receptivity and the abnormal contraction of the endometrium and its connected myometrium. Studies have shown that there is a high level of oxytocin (OT) in the serum of endometriosis (4). Previous studies have shown that OT acts on oxytocin receptor (OTR) in the uterine junction zone, activates the Ca^2+^ sub channel and releases Ca^2+^, thus achieving the effect of uterine smooth muscle contraction, and also stimulates the production and release of arachidonic acid and prostaglandin F_2a_ (PGF_2a_), thus stimulating uterine contraction, which may be the main reason for the strong peristalsis and dysperistalsis of endometrium in endometriosis (5). Accordingly, inhibiting uterine contraction has become an important method for increasing the implantation of embryos in patients with endometriosis and reducing pregnancy loss.

Phloroglucinol is a non-atropine, non-papaverine pure smooth-muscle antispasmodic, which can reduce abnormal contraction of uterine smooth muscle, thereby helping to evade the risk of abortion (6, 7). Considering that age is an independent factor affecting the success rate of embryo transfer, the effect of administering phloroglucinol to endometriosis patients at different ages, before and after frozen embryo transfer on their clinical pregnancy outcomes, was retrospectively analyzed herein to provide a clinical basis for improving the assisted pregnancy outcomes among these patients.

## Materials and method

### Participants

The clinical data of infertile patients with endometriosis who had undergone frozen embryo transfer (FET) in the reproductive center of the authors' hospital from August 2018 to August 2021 were collected.

According to the guidelines for the diagnosis and treatment of endometriosis of the Endometriosis Assistance Group of the Society of Obstetrics and Gynecology of the Chinese Medical Association, the diagnosis procedure of endometriosis is as follows (8):

(1) Clinical symptoms: dysmenorrhea, chronic pelvic pain, sexual intercourse pain, gastrointestinal symptoms related to the menstrual cycle, urinary symptoms related to the menstrual cycle, infertility with at least one of the above symptoms. Those with one or more of the above symptoms can be clinically diagnosed as endometriosis.
(2) The typical signs of endometriosis are retroverted fixation of the uterus, palpable cystic masses in the adnexal area, painful nodules in the posterior vaginal fornix, the rectouterine recess or the uterosacral ligament, or purplish blue nodules in the posterior vaginal fornix. Gynecological examination is helpful to the auxiliary diagnosis of endometriosis, especially deep infiltrating endometriosis.
(3) Imaging examination revealed ovarian endometriosis cyst.
(4) Serum CA_125_ is normal or increased.
(5) Laparoscopic examination revealed pelvic endometriosis cyst or nodule.

Endometriosis can be diagnosed by laparoscopic surgery or according to the patient's symptoms, medical history and (or) the results of gynecological examination, ultrasound examination and serological examination. At present, laparoscopy is considered as the golden standard for diagnosing endometriosis. The reliable and comprehensive diagnosis can be obtained by combining various means and confirming each other.

**The study's inclusion criteria were as follows**: (1) patients aged between 20 and 45 years; (2) patients with a body mass index (BMI) of 18.5–27.9 kg/m^2^; (3) patients who met the diagnosis of endometriosis (8); (4) patients whose endometrial preparation scheme was a down-regulation scheme of gonadotropin-releasing hormone agonist (GnRH-a); (5) patients with serum cancer antigen 125 (CA_125_) levels <35 IU/L on the day of hormone replacement; (6) patients with an endometrial thickness ≥8 mm on progesterone conversion; (7) patients transplanted with 1 high-quality blastocyst or 1–2 cleavage stage embryos, of which at least one was a high-quality embryo.

**The study's exclusion criteria were as follows**: (1) patients with abnormal uterine anatomy (e.g., a single, double, or residual-horned uterus, a uterine septum); (2) patients with a medical history of pelvic tuberculosis and hydrosalpinx; (3) patients with hysteromyoma ≥2 cm, endometrial polyps, and untreated intrauterine adhesions; (4) patients suffering from endocrine diseases (e.g., untreated diabetes, hyperthyroidism, hypothyroidism, hyperprolactinemia); (5) patients indicating embryo transfer difficulty (i.e., the embryo transfer tube showed significant resistance to entering the uterine cavity, the surgical process took a long time, and a probe or cervical dilator was needed); (6) patients whose spouse had abnormal karyotype test results (except chromosomal polymorphism); (7) patients who have not been pregnant after 3 or more embryo transfers.

**Figure 1:**
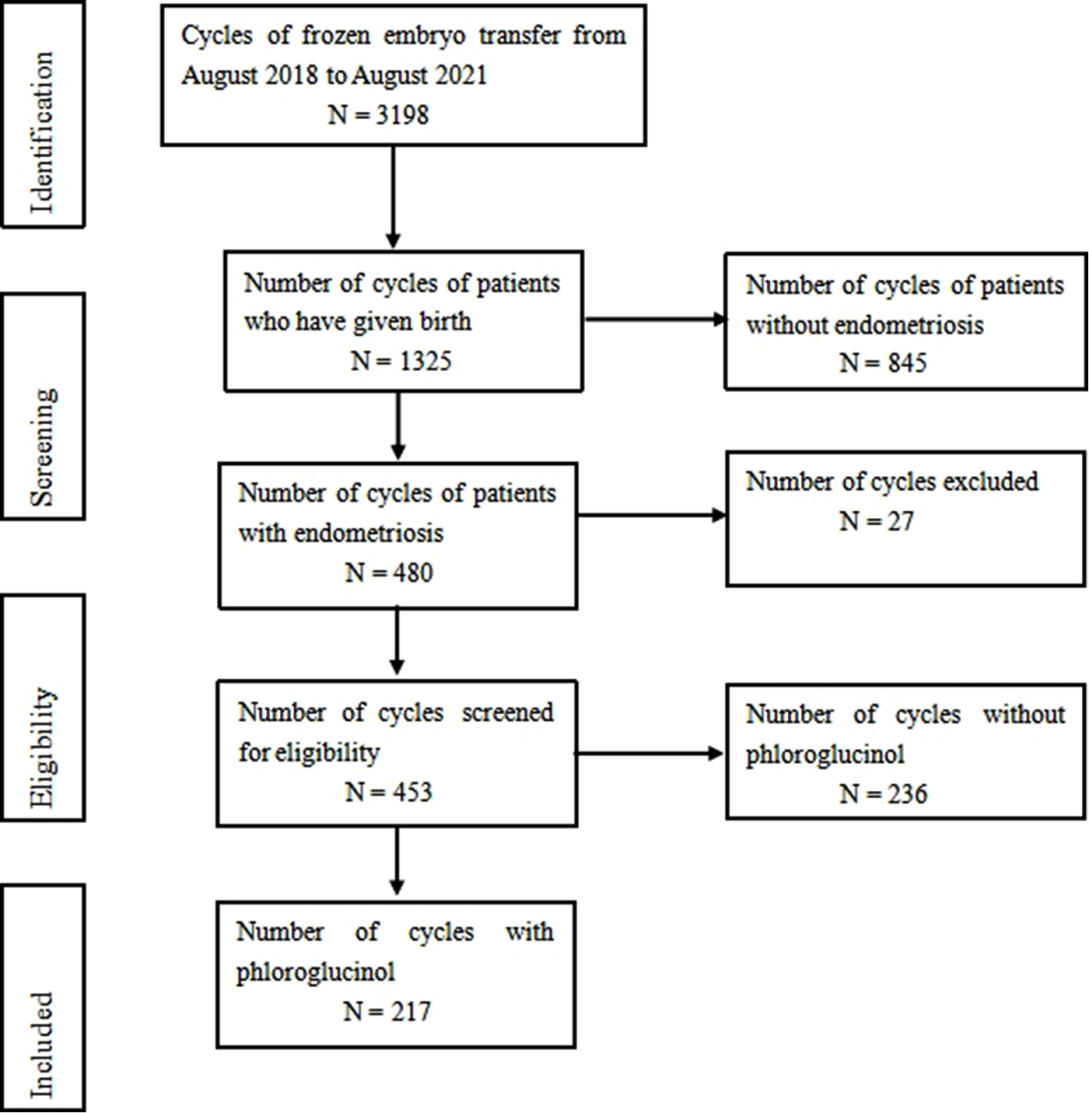
From August 2018 to August 2021, 3198 cycles of frozen embryo transfer were screened, including 1325 cases of delivery and 480 cases of endometriosis. According to the inclusion and exclusion criteria, 453 cases met the inclusion criteria, including 217 cases with phloroglucinol and 236 cases without phloroglucinol.

A total of 326 patients, 453 frozen embryo transfer cycles were incorporated in this study, including 217 cases in the phloroglucinol and 236 cases in the control groups, respectively (Figure 1). All patients completed a signed informed consent form before the administration of phloroglucinol. The control group was not treated with phloroglucinol.

Considering that age is an independent factor affecting the success rate of embryo transfer, the age of 35 years was used as a margin for dividing the female patients into a <35-year-old and a ≥35-year-old group, respectively, for observation of the effect of phloroglucinol on the pregnancy outcomes of embryo transfer for endometriosis patients of different ages. This study was approved by the Medical Ethics Committee of Shanghai Changzheng Hospital (20220121) and complied with the requirements set out in the Declaration of Helsinki.

## Study methods

### Ovarian stimulation and oocyte retrieval

Ovulation induction selection antagonist protocol: the recombinant follicle stimulating hormone (rFSH) 150–225 IU (Gonal-f, Merck Seranol Co., Ltd., Switzerland) was administered on the second or third day of the menstrual cycle. When the diameter of the dominant follicle reached 14 mm, 0.25 mg of gonadotropin releasing hormone antagonist (GnRH-A) was added daily (Cetrotide, Merck Seranol Co., Ltd., Switzerland) until the HCG day. Ovulation was triggered by 250 μg r-hCG (Merck Seranol Co., Ltd., Switzerland). When at least one leading follicle reached 18 mm or 2 reached 17 mm, the transvaginal ultrasound-guided follicle aspiration was performed to obtain the oocytes 36 h thereafter. Approximately 4–6 h after follicular aspiration, standard fertilization *in vitro* or intracytoplasmic sperm injection was performed as appropriate.

### Frozen embryo transfer preparation scheme

A GnRH-a down-regulation scheme was adopted, and 1–3 injections of triptorelin acetate (3.75 mg; Ipsen Pharma Biotech) were administered on day 2–3 of menstruation. Hormone replacement therapy can be used to prepare the endometrium after 28 days. Oral estradiol tablets (2 mg Fenmotilium; Abbott Biologics B.V.) were prescribed at 2–4 mg/day, as well as aspirin enteric-coated tablets (Bayer Schering Pharma) at 50–100 mg/day. The thickness of the endometrium and serum estradiol (E2) were observed by ultrasound on day 11 after treatment, and the maximum dosage of estradiol tablets was maintained at 6 mg. After 14–16 days of continuing the noted medications, when the endometrial thickness was ≥8 mm, E2 was >100 pg/ml, and progesterone (*P*) was <1.0 ng/ml, progesterone injection 40 mg (Zhejiang Xianju Pharmaceutical Co., Ltd.), intramuscular injection, once a day, until the date of embryo transfer. In this study, the low-dose aspirin was used to prevent the risk of venous thrombosis caused by long-term high-dose oral estrogen (9). The day when progesterone injection was started was taken as P0; the transplanting of 1–2 cleavage stage embryos occurred on *P* + 4 days, at least one of which was a high-quality embryo; the transplanting of one high-quality blastocyst took place on *P* + 6 days (according to Gardner's scoring standard (10), high-quality cleavage stage embryos were defined as follows: a cleavage number >6 cells on day 3 of fertilization, double pronuclear fertilization, fragmentation index <20%; a “high-quality blastocyst” referred to a stage 3 or above blastocyst, and the score of the inner cell mass and trophoblast was better than level C of this standard). Patients with history of spontaneous abortion accompanied with abnormal immune indicators or abnormal prethrombotic status related indicators, such as antinuclear antibody, anticardiolipin antibody, anti *β*^2^ glycoprotein antibody, anti double stranded DNA antibody, lupus antibody, were treated by low-dose immunomodulator or low molecular weight heparin.

### Grouping and treatment methods

Phloroglucinol was given to patients with dysmenorrhea or pelvic pain after previous embryo transfer. Patients were divided into two groups according to whether phloroglucinol was administered before and after the embryo transfer. For the phloroglucinol group, phloroglucinol (40 mg; Nanjing Hengsheng Pharmaceutical Co., Ltd.) was injected intramuscularly 30 min before surgery on the day of embryo transfer and once a day from the date of progesterone administration up to day 7 after embryo transfer. The control group was not treated with phloroglucinol. Because age is an independent factor that affects ovarian reserve function and the success rate of an embryo transfer, the age of 35 was used as the boundary to divide the female patients into a <35 and ≥35-year-old group, respectively.

### Corpus luteum support

Estradiol and dydrogesterone tablets (Femostone yellow tablets) were prescribed for both groups at 6 mg/day; one progesterone vaginal sustained-release gel (Crinone, 90 mg [8%]; Fleet Laboratories Limited) was administered vaginally once daily, and aspirin enteric-coated tablets were maintained at the dose noted previously. For patients with a prethrombotic state and rheumatic immune diseases, the originally related medications should be maintained.

### Evaluation criteria

“Biochemical pregnancy” was defined when the value of human chorionic gonadotropin detected on day 14 after the embryo transfer was ≥10 U/L; on days 28–35 after the embryo transfer, an intrauterine clinical pregnancy was considered if the gestational sac could be observed in the uterine cavity through transvaginal color Doppler ultrasound examination; in this instance, the corpus luteum support drug was used until 8–10 weeks after embryo transfer. If the gestational sac was outside the uterine cavity, pregnancy was defined as being ectopic. Spontaneous abortion before 12 weeks of gestation was considered an early abortion.

### Outcome measures

The current study applied the following outcome measures:

• The biochemical pregnancy rate = the number of biochemical pregnancy cycles/the number of embryo transfer cycles × 100%.
• The embryo implantation rate = the number of intrauterine gestational sacs/the number of transferred embryos × 100%.
• The clinical pregnancy rate = the number of clinical pregnancy cycles/the number of embryo transfer cycles × 100%.
• The ectopic pregnancy rate = the number of ectopic pregnancy cycles/the number of embryo transfer cycles × 100%.
• The early abortion rate = the number of spontaneous abortion cases within 12 weeks of gestation/the number of clinical pregnancy cycles × 100%.
• The live birth rate = the number of patients with live birth/the number of embryo transfer cycles × 100%.

### Statistical analysis

The SPSS Statistics 22.0 software was used to conduct statistical analysis. The measurement data were expressed by the mean and standard deviation (*х*), and the independent sample t-test was used. The data were expressed as a percentage (%), and the chi-square (*χ*^2^) test was used to checked these percentages. The two-sided test was adopted, with the test level alpha established as 0.05, and *P* < 0.05 was considered to be statistically significant.

## Results

### General data of the two patient groups

A total of 453 cycles were screened for the included cases, including 217 in the phloroglucinol group and 236 in the control group. The basic data characteristics of the two patient groups are shown in Table 1. After conducting statistical analysis and comparisons, no statistical significance was evident in terms of the age (*P* = 0.707), duration of infertility (*P* = 0.912), BMI (*P* = 0.589), primary infertility and secondary infertility (*P* = 0.763), intima thickness on the day of embryo transfer (*P* = 0.054), the number of transplanted embryos (*P* = 0.985), or the developmental stage of the transferred embryos (*P* = 0.095) among the different groups.

**Table 1:** Comparison between two groups of patients in terms of general information.

Items	Phloroglucinol group (*n* = 217)	Control group (*n* = 236)	*P*
Age (years)	33.62 ± 4.69	33.79 ± 4.91	0.707
Infertility duration (years)	3.92 ± 2.51	3.89 ± 2.52	0.912
BMI (kg/m^2^)	21.85 ± 2.18	21.73 ± 2.26	0.589
Proportion of primary infertility	45.6 (99/217)	47.03 (111/236)	0.763
Proportion of secondary infertility	54.38 (118/217)	52.97 (125/236)	
Causes of infertility			
Fallopian tube factor	56.22 (122/217)	54.24 (128/236)	0.671
Ovulation disorder	38.25 (83/217)	41.95 (99/236)	0.422
Male factor	5.53 (12/217)	3.81 (9/236)	0.385
Pregnancy history			
Gravida	3.0 (0–6)	3.5 (0–7)	0.103
Para	1 (0–2)	1 (0–2)	0.527
ET failure times	1.12 ± 0.84	1.24 ± 0.76	0.133
Endometrial thickness on the day of embryo transfer (mm)	10.31 ± 2.00	9.94 ± 2.04	0.054
Mean number of transferred embryos	1.59 ± 0.49	1.59 ± 0.51	0.985
Mean number of high quality embryos	1.49 ± 0.50	1.49 ± 0.50	0.902
**Developmental stage of the transferred embryos**			
Cleavage stage embryos	81.11 (176/217)	74.58 (176/236)	0.095
Branstocysts	18.89 (41/217)	25.42 (60/236)	

### Comparison between the two patient groups concerning the clinical outcomes

The biochemical pregnancy rate (*P* = 0.011), embryo implantation rate (*P* = 0.002), clinical pregnancy rate (*P* = 0.018), and live birth rate (*P* = 0.001) in the phloroglucinol group were higher than in the control group. The early abortion rate in the phloroglucinol group was statistically lower compared to the control group (*P* = 0.005) (see Table 2).

**Table 2:** Comparison between two groups in terms of pregnancy outcomes (%).

	Phloroglucinol group	Control group	*χ* ^2^	*P*
Biochemical pregnancy rate	63.13 (137/217)	51.27 (121/236)	6.489	0.011
Embryo implantation rate	44.64 (154/345)	33.60 (126/375)	9.211	0.002
Clinical pregnancy rate	59.44 (129/217)	48.30 (114/236)	5.644	0.018
Ectopic pregnancy rate	1.55 (2/129)	3.51 (4/114)	0.322	0.570*
Early abortion rate	7.75 (10/129)	20.18 (23/114)	7.959	0.005
Live birth rate	52.99 (115/217)	36.86 (87/236)	11.906	0.001

* Theoretical frequency is less than 5, and the continuous correction *χ*^2^ was used for test.

### Comparison between the different patient age groups concerning general information

Since age is an independent factor affecting the success rate of pregnancy, the age of 35 was used as the limit for dividing the female patients into a <35 and ≥35-year-old group, respectively, including 137 patients in the phloroglucinol group and 139 in the control group. The ≥35-year-old group included 80 patients in the phloroglucinol group and 97 in the control group. The basic data characteristics of the two patient groups are shown in Table 3. After conducting statistical analysis and comparisons, no statistical significance was found in the age (*P* = 0.835), duration of infertility (*P* = 0.928), BMI (*P* = 0.644), primary infertility and secondary infertility (*P* = 0.666), intima thickness on the day of embryo transfer (*P* = 0.282), the number of transplanted embryos (*P* = 0.985), and the developmental stage of the transferred embryos (*P* = 0.390) among the different groups.

**Table 3:** Comparison between different Age groups of patients in terms of general information.

	<35-year-old group		*P*	≥35-year-old group		*P*
	Phloroglucinol group (*n* = 137)	Control group (*n* = 139)		Phloroglucinol group (*n* = 80)	Control group (*n* = 97)	
Age (years)	30.64 ± 2.51	30.41 ± 2.82	0.471	38.73 ± 2.77	38.64 ± 2.69	0.835
Infertility duration (years)	3.44 ± 2.00	3.25 ± 2.36	0.480	4.75 ± 3.04	4.80 ± 4.57	0.928
BMI (kg/m^2^)	21.49 ± 1.99	21.34 ± 2.17	0.555	22.45 ± 2.35	22.29 ± 2.27	0.644
Proportion of primary infertility	60.58 (83/137)	64.03 (89/139)	0.555	20 (16/80)	22.68 (22/97)	0.666
Proportion of secondary infertility	39.42 (54/137)	35.97 (50/139)		80 (64/80)	77.32 (75/97)	
Endometrial thickness on the day of embryo transfer (mm)	10.37 ± 2.00	10.02 ± 1.71	0.115	10.21 ± 2.02	9.84 ± 2.44	0.282
Mean number of transferred embryos	1.62 ± 0.49	1.63 ± 0.50	0.927	1.54 ± 0.50	1.54 ± 0.52	0.985
Mean number of high quality embryos	1.54 ± 0.50	1.51 ± 0.50	0.627	1.41 ± 0.50	1.45 ± 050	0.585
Developmental stage of the transferred embryos						
Cleavage stage embryos	78.10 (107/137)	69.78 (97/139)	0.116	86.25 (69/80)	81.44 (79/97)	0.390
Branstocysts	21.90 (30/137)	30.22 (42/139)		13.75 (11/80)	18.56 (18/97)	

### Comparison between different patient age groups concerning clinical outcomes

For patients aged <35 years, the embryo implantation (*P* = 0.008), clinical pregnancy (*P* = 0.042), and live birth rates (*P* = 0.002) in the phloroglucinol group were higher than in the control group, and the early abortion rate was lower compared with the control group. The differences were statistically significant. For patients aged ≥35 years, no ectopic pregnancy occurred in either the phloroglucinol or control group, and no significant difference was observed in the embryo transfer (*P* = 0.209), clinical pregnancy (*P* = 0.311), early abortion (*P* = 0.217), and live birth rates (*P* = 0.182) (see Table 4).

**Table 4:** Comparison of pregnancy outcomes of patients in different Age groups (%).

	<35-year-old group		*χ* ^2^	*P*	≥35-year-old group		*χ* ^2^	*P*
	Phloroglucinol group (*n* = 137)	Control group (*n* = 139)			Phloroglucinol group (*n* = 80)	Control group (*n* = 97)		
Biochemical pregnancy rate	69.34 (95/137)	61.87 (86/139)	1.707	0.191	52.5 (42/80)	36.08 (35/97)	4.808	0.028
Embryo implantation rate	51.81 (115/222)	39.38 (89/226)	6.967	0.008	31.71 (39/123)	24.83 (37/149)	1.582	0.209
Clinical pregnancy rate	69.34 (95/137)	57.55 (80/139)	4.133	0.042	42.5 (34/80)	35.05 (34/97)	1.028	0.311
Ectopic pregnancy rate	2.11 (2/95)	5 (4/80)	0.399	0.528*	0	0	/	/
Early abortion rate	6.32 (6/95)	17.5 (14/80)	5.367	0.021	11.76 (4/34)	26.47 (9/34)	1.522	0.217*
Live birth rate	63.50 (87/137)	44.60 (62/139)	9.921	0.002	35 (28/80)	25.77 (25/97)	1.779	0.182

* Theoretical frequency is less than 5, and the continuous correction 2 was used for test.

## Discussion

To date, more than 8 million babies have been born worldwide as a result of IVF treatment (11). However, in the past four decades, the clinical outcomes of reproductive technology-assisted pregnancy have been unsatisfactory, with the live birth rate per cycle being only approximately 30% (12). Good embryo quality, endometrial receptivity, and synchronization of the endometrium and embryo development can provide the necessary conditions for the successful implantation of an embryo (13). If the uterine environment is abnormal, two-thirds of all pregnancies will fail (14).

Endometriosis is a common gynecological disease in women of childbearing age, and 30%–50% of women with endometriosis suffer from infertility (2), which may be related to impaired endometrial receptivity resulting from inflammation and immune abnormalities brought on by endometriosis itself, ultimately resulting in the abnormal contraction of the myometrium (5). Studies (15) have confirmed that ascites from patients with endometriosis contained high concentrations of prostaglandins (PGs), which stimulate uterine contraction and lead to abnormal tubal peristalsis, causing the arrival time of the fertilized egg in the uterine cavity to be out of sync with endometrial development, thereby affecting the implantation of fertilized eggs. In addition, during the embryo implantation period, ultrastructural changes occur in the endometrium, such as a lack of the expression of key factors, e.g., integrin, selectin, and cadherin, resulting in decreased endometrial receptivity. Under normal circumstances, the endometrium can generate endometrial peristaltic waves with non-synchronous contraction of the submucosal layer of the endometrium. The frequency of uterine contraction will be gradually reduced after ovulation and will steadily disappear during the implantation window period, which is conducive to the adhesion, positioning, and implantation of the embryo in the uterine cavity (16). However, during IVF-assisted pregnancy, some researchers have observed that a frequency <3 times/min of uterine contraction occurred less than 25% before embryo transfer but could reach 34% >5 times/min (17). Moreover, studies have found that about one-third of patients will have uterine contractions with a frequency of more than 5 times/minute on the day of embryo transfer, while too frequent uterine contractions will push the embryo to the fallopian tube or cervix, or even push the embryo out of the uterine cavity, leading to pregnancy failure (18, 19). As such, excessive uterine smooth muscle contraction and abnormal endometrial movement modes and frequency can lead to embryo implantation failure, early pregnancy loss, and even ectopic pregnancy (20).

Based on the above, inhibiting uterine contraction has become an important means of increasing embryo implantation and reducing pregnancy loss in infertile patients with endometriosis. At present, commonly used uterine contraction inhibitors include progesterone, oxytocin antagonist, and PG antagonist (21). Phloroglucinol is a myopathic non-atropine non-papaverine smooth muscle antispasmodic with 1,3,5-trihydroxybenzene as the active ingredient (6), which has no anticholinergic effect, no effect on cardiovascular function, does not cause hypotension, has no teratogenicity, mutagenicity, or carcinogenicity, and no adverse effect on embryo and fetal development (22). In one study, researchers administered 80 mg phloroglucinol intravenously twice a day to IVF-ET-assisted pregnancy patients up to 5 days before embryo transfer, trusting that it could reduce the uterine contraction of patients and increase the clinical pregnancy rate (23). Another study posited that repeat visits for medication are inconvenient for patients; therefore, only 80 mg of phloroglucinol was given intravenously before the embryo transfer, which also led to a good clinical pregnancy outcome (24).

In the above studies, phloroglucinol was only given for a single or multiple times during the embryo transfer period, but according to the law that uterine contraction drives endometrial peristalsis, in the natural state, with the development of follicles, the endometrial peristalsis from the cervix to the fundus increases, and reaches the peak before ovulation, which is conducive to sperm delivery. After ovulation, the peristalsis of endometrium is significantly reduced, forming a quiet and stable environment, which makes it possible for embryo implantation (25). Moreover, in this study, it was observed that endometrial peristalsis in endometriosis patients began to increase on the day of progesterone application, and some patients had uterine contraction symptoms within one week after embryo transfer, such as abdominal distension and dysmenorrhea, especially in the afternoon. Therefore, we gave continuous uterine contraction inhibition during the period of frequent endometrial peristalsis, and started to give phloroglucinol intramuscular injection in the afternoon of progesterone application day, once a day, until 7 days after embryo transfer. During embryo transfer, the dosage of phloroglucinol is still not uniform. Considering that phloroglucinol has a fast onset, short half-life, and the half-life of blood concentration is about 15 min, the blood concentration decreases rapidly within 4 h after administration, and then slowly decreases. After 48 h, there is only a small amount of drug residue in the body (26). In addition, previous literature reported that phloroglucinol 40 mg was injected intramuscularly, it also has a positive effect on improving the clinical pregnancy outcome of patients with repeated embryo implantation failure (27). Therefore, based on the principle of low dose, safety and effectiveness, we tried to use phloroglucinol at a dose of 40 mg.

The results of this study showed that continuous low-dose phloroglucinol pretreatment before and after embryo transfer could significantly improve the implantation, clinical pregnancy, and live birth rates of endometriosis patients by inhibiting excessive endometrial peristalsis and uterine smooth muscle contraction from ovulation to mid luteal phase, and the results were consistent with existing studies (23, 24). Moreover, the results of a meta-analysis in 2019 (7) showed that phloroglucinol had advantages in the overall response rate to the threat of abortion, the time to relieve uterine contraction, and the symptoms of uterine contraction; the same results were obtained in the current study. In addition, during phloroglucinol intervention, these patients had no complaints and experienced no allergic reactions.

Considering age as an independent factor affecting the success rate of pregnancy, the age of 35 was used as a boundary for dividing the patients with endometriosis into <35 and ≥35-year-old groups, respectively. The results showed that in young patients, continuous small-dose administration of phloroglucinol before and after embryo transfer could significantly improve pregnancy outcomes. However, for the older patient group, although the embryo implantation, clinical pregnancy, and live birth rates after phloroglucinol pretreatment were higher than in the control group, no statistical difference was observed. This indicated the presence of several factors affecting the blastocyst implantation in older patients, and phloroglucinol could not improve the pregnancy outcome in these participants with endometriosis. In addition, phloroglucinol pretreatment did not reduce the abortion rate in older patients under the influence of an increased risk of fetal chromosomal abnormalities. Existing studies have shown that phloroglucinol pretreatment before embryo transfer could effectively inhibit the occurrence of ectopic pregnancy (23). In this study, although patients of different ages accounted for a relatively high proportion of the embryos in the cleavage stage of embryo transfer, the rates of ectopic pregnancy were low, and no significant differences were observed. It was considered that phloroglucinol may inhibit excessive peristalsis of the endometrium, thereby reducing the occurrence of an ectopic pregnancy.

In summary, the infertility caused by endometriosis is related to the over secretion of cytokines such as prostaglandins and metalloproteinases, which damages the functions of ovary, peritoneum, fallopian tube and endometrium, and leads to the limitation of follicle generation, fertilization and implantation (28). The widespread adhesion in the pelvic cavity will hinder the release of oocytes, prevent sperm from entering the abdominal cavity or inhibit the oviduct from collecting eggs, thus reducing fertility. The reason of infertility caused by moderate and severe endometriosis may be that the disease causes premature loss of ovarian follicle reserve, abnormal follicular development, and decreased fertilization potential of oocytes. While phloroglucinol directly acts on the smooth muscle of gastrointestinal tract and genitourinary tract (29). It is a myophilic, non-atropine, non-papaverine, pure smooth muscle spasmolytic drug, which can effectively reduce the abnormal contraction of uterine smooth muscle and maintain the normal function of fallopian tube and endometrium. Phloroglucinol, as a safe, effective, economical, and practical smooth muscle antispasmodic, could significantly inhibit abnormal uterine smooth muscle contraction in young patients and reduce excessive and frequent endometrial peristalsis, thereby improving clinical pregnancy outcomes. As such, this approach is anticipated to become a new technique for improving pregnancy outcomes among infertile patients with endometriosis. The present research was a retrospective study comprising a small sample size, however, and its results will require confirmation by multicenter clinical studies with larger sample sizes.

## Data Availability

The original contributions presented in the study are included in the article/Supplementary Material, further inquiries can be directed to the corresponding author/s.
